# Resilience to cognitive impairment in the oldest-old: design of the EMIF-AD 90+ study

**DOI:** 10.1186/s12877-018-0984-z

**Published:** 2018-11-26

**Authors:** Nienke Legdeur, Maryam Badissi, Stephen F. Carter, Sophie de Crom, Aleid van de Kreeke, Ralph Vreeswijk, Marijke C. Trappenburg, Mardien L. Oudega, Huiberdina L. Koek, Jos P. van Campen, Carolina J. P. W. Keijsers, Chinenye Amadi, Rainer Hinz, Mark F. Gordon, Gerald Novak, Jana Podhorna, Erik Serné, Frank Verbraak, Maqsood Yaqub, Arjan Hillebrand, Alessandra Griffa, Neil Pendleton, Sophia E. Kramer, Charlotte E. Teunissen, Adriaan Lammertsma, Frederik Barkhof, Bart N. M. van Berckel, Philip Scheltens, Majon Muller, Andrea B. Maier, Karl Herholz, Pieter Jelle Visser

**Affiliations:** 1 Alzheimer Center Amsterdam, Department of Neurology, Amsterdam Neuroscience, Vrije Universiteit Amsterdam, Amsterdam UMC, PO Box 7057, 1007 MB Amsterdam, the Netherlands; 2 Wolfson Molecular Imaging Centre, Division of Neuroscience & Experimental Psychology, University of Manchester, Manchester, UK; 3 Department of Ophthalmology, Amsterdam Neuroscience, Vrije Universiteit Amsterdam, Amsterdam UMC, Amsterdam, The Netherlands; 4 Department of Geriatric Medicine, Spaarne Gasthuis, Haarlem, The Netherlands; 5 Department of Internal Medicine, Amstelland Hospital, Amstelveen, The Netherlands; 6 Department of Psychiatry, Vrije Universiteit Amsterdam, Amsterdam UMC, Amsterdam, The Netherlands; 7 Department of Geriatric Medicine, University Medical Center Utrecht, Utrecht, The Netherlands; 8 Department of Geriatric Medicine, MC Slotervaart Hospital, Amsterdam, The Netherlands; 9 Department of Geriatric Medicine, Jeroen Bosch Hospital, ‘s-Hertogenbosch, The Netherlands; 10 Teva Pharmaceuticals, North Wales, Pennsylvania USA; 11 Janssen Pharmaceutical Research and Development, Titusville, NJ USA; 12 Boehringer Ingelheim International GmbH, Ingelheim/Rhein, Germany; 13 Department of Internal Medicine, Vrije Universiteit Amsterdam, Amsterdam UMC, Amsterdam, The Netherlands; 14 Department of Radiology & Nuclear Medicine, Amsterdam Neuroscience, Vrije Universiteit Amsterdam, Amsterdam UMC, Amsterdam, The Netherlands; 15 Department of Clinical Neurophysiology and MEG Center, Amsterdam Neuroscience, Vrije Universiteit Amsterdam, Amsterdam UMC, Amsterdam, The Netherlands; 16 Dutch Connectome Lab, Department of Complex Trait Genetics, Center for Neuroscience and Cognitive Research, Amsterdam Neuroscience, Vrije Universiteit Amsterdam, Amsterdam UMC, Amsterdam, The Netherlands; 17 Department of Otolaryngology-Head and Neck Surgery, Section Ear & Hearing, Amsterdam Public Health Research Institute, Vrije Universiteit Amsterdam, Amsterdam UMC, Amsterdam, The Netherlands; 18 Neurochemistry Laboratory, Department of Clinical chemistry, Amsterdam Neuroscience, Vrije Universiteit Amsterdam, Amsterdam UMC, Amsterdam, The Netherlands; 19 Institutes of Neurology and Healthcare Engineering, University College London, London, UK; 20 Department of Medicine and Aged Care, @AgeMelbourne, Royal Melbourne Hospital, University of Melbourne, Melbourne, Australia; 21 Department of Human Movement Sciences, @AgeAmsterdam, Amsterdam Movement Sciences, Vrije Universiteit Amsterdam, Amsterdam, The Netherlands; 22 Department of Psychiatry & Neuropsychology, School for Mental Health and Neuroscience, Maastricht University, Maastricht, The Netherlands

**Keywords:** Alzheimer’s disease, Dementia, Cognitive impairment, Amnestic mild cognitive impairment, Resilience, Oldest-old, Amyloid, Positron emission tomography, Magnetoencephalography (MEG)

## Abstract

**Background:**

The oldest-old (subjects aged 90 years and older) population represents the fastest growing segment of society and shows a high dementia prevalence rate of up to 40%. Only a few studies have investigated protective factors for cognitive impairment in the oldest-old. The EMIF-AD 90+ Study aims to identify factors associated with resilience to cognitive impairment in the oldest-old. In this paper we reviewed previous studies on cognitive resilience in the oldest-old and described the design of the EMIF-AD 90+ Study.

**Methods:**

The EMIF-AD 90+ Study aimed to enroll 80 cognitively normal subjects and 40 subjects with cognitive impairment aged 90 years or older. Cognitive impairment was operationalized as amnestic mild cognitive impairment (aMCI), or possible or probable Alzheimer’s Disease (AD). The study was part of the European Medical Information Framework for AD (EMIF-AD) and was conducted at the Amsterdam University Medical Centers (UMC) and at the University of Manchester. We will test whether cognitive resilience is associated with cognitive reserve, vascular comorbidities, mood, sleep, sensory system capacity, physical performance and capacity, genetic risk factors, hallmarks of ageing, and markers of neurodegeneration. Markers of neurodegeneration included an amyloid positron emission tomography, amyloid β and tau in cerebrospinal fluid/blood and neurophysiological measures.

**Discussion:**

The EMIF-AD 90+ Study will extend our knowledge on resilience to cognitive impairment in the oldest-old by extensive phenotyping of the subjects and the measurement of a wide range of potential protective factors, hallmarks of aging and markers of neurodegeneration.

**Trial registration:**

Nederlands Trial Register NTR5867. Registered 20 May 2016.

**Electronic supplementary material:**

The online version of this article (10.1186/s12877-018-0984-z) contains supplementary material, which is available to authorized users.

## Background

### Introduction

The oldest-old (subjects aged 90 years and older) population represents the fastest growing segment of society [1]. Worldwide, the number of oldest-old subjects is expected to increase to 71.2 million in 2050, a 5-fold increase of the current oldest-old population [2, 3]. The oldest-old have a high risk of developing dementia with a prevalence up to 40% [4]. The increasing number of oldest-old subjects with dementia will have major clinical and financial consequences for patients, their families and society as a whole [5].

Still a considerable number of subjects remain cognitively normal at high age, indicating the presence of protective factors for cognitive impairment in these subjects. Identification of these protective factors is crucial and will have implications for preventive strategies. In addition, identifying the neurodegenerative markers associated with cognitive impairment in the oldest-old, will enhance our understanding of the underlying pathophysiology in this specific age group.

The EMIF-AD 90+ study was set-up to investigate protective factors for cognitive impairment in the oldest-old. We will first provide an overview of the current status of research on this topic and then present the study outline of the EMIF-AD 90+ study.

### Review on studies on cognitive impairment in the oldest-old

We searched for studies focusing on protective factors for cognitive impairment in nonagenarians, which gave us two results: ***The 90+ Study*** in the USA and the Danish Birth Cohort Studies [6, 7]. Broadening the search to studies that started inclusion from the age of 85 years or focused on successful aging resulted in eight more studies: the H85 Gothenburg study, Leiden 85-plus Study, Newcastle 85+ Study, NonaSantfeliu study, Octabaix study, Project of Longevity and Aging in Dujangyan (PLAD), Umeå study and Vantaa 85+ Study [8–15]. Table 1 shows the design characteristics of these ten studies.

**Table 1:** Design characteristics of other 85+ and 90+ studies that include data about cognitive functioning

Domain	Danish Birth Cohort Studies [6]^a^	H85 Gothen-burg study [12]	Leiden 85-plus Study [9]	Newcastle 85+ Study [10]	NonaSant-feliu study [8]	Octabaix study [13]	PLAD [15]	The 90+ Study, USA [7]	Umeå 85+ study [14]	Vantaa 85+ Study [11]
Cognitive reserve	+	+	+	+	+	+	+	+	+	+
Vascular comorbidity	+	+	+	+	+	+	+	+	+	+
Mood and sleep	+	+	+	+	–	–	+	+	+	+
Sensory system	–	–	+	+	+	+	–	+	+	–
Physical performance and capacity	+	–	+	+	–	+	+	+	+	–
Genetics	+	+	+	+	–	–	+	+	–	+
Hallmarks of aging^b^	–	–	+	–	–	+	–	+	–	–
Markers of neurodegeneration	–	+	–	–	–	–	–	+	–	–

*PLAD* Project of Longevity and Aging in Dujangyan

^a^Including the cohorts recruited in 1895, 1905, 1910 and 1915, data availability varies per cohort. ^b^Inflammation and senescence markers (for example p16, p53 and telomere associated foci)

#### Protective factors for cognitive impairment in the oldest-old

Table 2 summarizes the findings on the protective factors for cognitive impairment or dementia of the ten studies. A high level of education was found to be protective against dementia in the oldest-old and one study indicated that high cognitive activity, examined by looking at the time spend on reading, around age 90 years was related to resilience to dementia [4, 16–18]. The influence of vascular comorbidities on cognition has been studied quite extensively in this age group. Most studies did not find an association between cholesterol levels and cognition in the oldest-old [15, 17, 19–22]. Hypertension has mostly been found to be protective in the oldest-old, especially when hypertension is diagnosed after the age of 80 years [17, 19, 23–27]. This is in contrast to studies that have shown a higher dementia risk in the presence of midlife hypertension [28]. In addition, although midlife diabetes mellitus has been related to dementia in younger subjects [29], the influence of diabetes mellitus on cognition might be less evident in the oldest-old [11, 30, 31]. The protective effect related to the absence of stroke seemed to persist in the oldest-old [18, 32] and one study on atrial fibrillation and dementia did not find an association [32]. The absence of depressive symptoms seemed to be associated with resilience to cognitive impairment, which is consistent with findings in younger subjects [14, 33, 34]. One study related sleep quality to cognition and reported a higher sleep quality in subjects without cognitive impairment, which is in line with results in younger subjects [35, 36]. With regard to the sensory system, visual and auditory impairments have been associated with worse cognitive functioning in the oldest-old [37, 38] and although olfactory impairment has been associated with incident dementia in a younger age group [39], no studies were found studying this in the oldest-old.

**Table 2:** Potential protective factors for cognitive impairment in the oldest-old

Domain	Potential protective factor	Study	Age^a^	Sample size (N)	Outcome variable	Result
Cognitive reserve	High level of education	H85 Gothenburg study [18]	85.7 (±0.05)	No dementia: 794 Dementia: 271	Dementia	Protective
		The 90+ Study [4]	94 (90–106)	No dementia: 536 Dementia: 375	Dementia	Protective
		Vantaa 85+ Study [17]	88.4 (85.0–104.0)	No incident dementia: 239 Incident dementia: 100	Incident dementia	Protective
	High cognitive activity	The 90+ Study [16]	93 (90–103)	No incident dementia: 319 Incident dementia: 268	Incident dementia	Equivocal
Vascular comorbidity	Low total/LDL or high HDL cholesterol level	Leiden 85-plus Study [20]	85 (85)	No dementia: 488 Dementia: 73	Cognition Dementia	Equivocal
		Newcastle 85+ Study [19]	85 (85)	No dementia: 767 Dementia: 78	Cognition Cognitive decline	Equivocal
		NonaSantfeliu study [21]	94.3 (±2.6)	62, dementia status unknown	Cognition	No effect
		Octabaix study [22]	85 (85)	321, dementia status unknown	Cognition	No effect
		PLAD [15]	93.6 (90–108)	No cognitive impairment: 300 Cognitive impairment: 409	Cognition	No effect
		Vantaa 85+ Study [17]	88.4 (85.0–104.0)	No incident dementia: 239 Incident dementia: 100	Incident dementia	No effect
	Absence of hypertension	Leiden 85-plus Study [23]	85 (85)	572, dementia status unknown	Cognition Cognitive decline	Risk
		Newcastle 85+ Study [19]	85 (85)	No dementia: 767 Dementia: 78	Cognition Cognitive decline	Equivocal
		PLAD [27]	93.6 (90–108)	No cognitive impairment: 317 Cognitive impairment: 465	Cognition	No effect
		Umeå 85+ study [26]	85, 90 and ≥ 95	No dementia: 342 Dementia: 233	Cognition Dementia	Protective
		Umeå 85+ study [25]	88.8 (±4.1)	No incident dementia: 136 Incident dementia: 69	Incident dementia	No effect
		The 90+ Study [24]	93.2 (90–103)	No incident dementia: 335 Incident dementia: 224	Incident dementia	Risk
		Vantaa 85+ Study [17]	88.4 (85.0–104.0)	No incident dementia: 239 Incident dementia: 100	Incident dementia	Equivocal
	Absence of DM	Leiden 85-plus Study [30]	85 (85)	596, dementia status unknown	Cognition Cognitive decline	Equivocal
		Octabaix study [31]	85 (85)	167, dementia status unknown	Cognition Cognitive decline	No effect
		Vantaa 85+ Study [11]	≥85	No incident dementia: 249 Incident dementia: 106	Incident dementia	Protective
	Absence of stroke	H85 Gothenburg study [18]	85.7 (±0.05)	No dementia: 794 Dementia: 271	Dementia	Protective
		Vantaa 85+ Study [32]	88.4 (±2.9)	No dementia: 339 Dementia: 214 Incident dementia: 100	Dementia Incident dementia	Protective
	Absence of AF	Vantaa 85+ Study [32]	88.4 (±2.9)	No dementia: 339 Dementia: 214 Incident dementia: 100	Dementia Incident dementia	No effect
Mood and sleep	No depression	Leiden 85-plus Study [34]	85 (85)	500, dementia status unknown	Cognition	Protective
		Umeå 85+ study [14]	85, 90 and 95–103	No dementia: 173 Dementia: 69	Dementia	Protective
	High sleep quality	PLAD [35]	93.5 (±3.4)	No dementia: 251 Dementia: 409	Dementia Cognition	Protective
Sensory system	Absence of visual impairment	Leiden 85-plus Study [37]	85 (85)	459, dementia status unknown	Cognition	Protective
		Newcastle 85+ Study [38]	85 (85)	No dementia: 771 Dementia: 68	Cognition	Protective
	Absence of glaucoma or cataract	Newcastle 85+ Study [105]	85 (85)	No dementia: 771 Dementia: 68	Cognition	Equivocal
	Absence of hearing impairment	Leiden 85-plus Study [37]	85 (85)	459, dementia status unknown	Cognition	Equivocal
Physical performance and capacity	Good physical performance	Leiden 85-plus Study [40]	85 (85)	555, dementia status unknown	Cognition	Protective
		The 90+ Study [41]	93.3 (±2.6)	No incident dementia: 366 Incident dementia: 212	Incident dementia	Protective
	High physical activity	The 90+ Study [16]	93 (90–103)	No incident dementia: 319 Incident dementia: 268	Incident dementia	No effect
Genetics	Absence of APOEε4 and/or presence of APOEε2	Danish 1905 birth cohort [42]	93.1 (±0.3)	1551, dementia status unknown	Cognition Cognitive decline	No effect
		Leiden 85-plus Study [43]	89.0 (87.4–91.2)^b^	No dementia: 242 Dementia: 78	Dementia	Protective
		The 90+ Study [44]	93.7 (90–105)	No dementia: 566 Dementia: 236 Incident dementia: 188	Dementia Incident dementia	Equivocal
		Vantaa 85+ Study [45]	≥85	313 without dementia 197 with dementia	Dementia	Protective
		Vantaa 85+ Study [46]	≥85	No incident dementia: 187 Incident dementia: 58	Incident dementia Cognitive decline	No effect
	MnSOD, GLRX, GSTP1, MT1A, NDUFV1, PRDX3, UQCRFS1, PICALM	Danish 1905 birth cohort [106–108]	92-93^c^	1089–1650, dementia status unknown	Cognition	Protective
	ACOX1	Danish 1905 birth cohort [106]	93.2 (92.7–93.8)	1089, dementia status unknown	Cognition	Risk
	Cytokine genes, CLU	Danish 1905 birth cohort [108–110]	92-93^c^	1380–1651, dementia status unknown	Cognition Cognitive decline	Equivocal
	MTHFR, MTR	Danish 1905 birth cohort [111]	93.1 (±0.3)	1651, dementia status unknown	Cognition Cognitive decline	No effect
	KLOTHO	PLAD [112]	93.5 (90–108)	No cognitive impairment: 236 Cognitive impairment: 470	Cognition	Protective
	PPAR-γ2	PLAD [113]	93.7 (90–108)	No cognitive impairment: 257 Cognitive impairment: 475	Cognition	No effect
	LRP, LPL, ACE	Vantaa 85+ Study [114]	≥85	No dementia: 203 Dementia (AD): 113	Dementia	No effect
Hallmarks of ageing	Low level of inflammation markers	Leiden 85-plus Study [49]	85 (85)	No dementia: 491	Cognition Cognitive decline	Equivocal
		The 90+ Study [50]	94.3 (90–105)	No dementia: 232 Dementia: 73	Dementia	Equivocal
		The 90+ Study [51]	93.9 (90–102)	No incident dementia: 145 Incident dementia: 82	Incident dementia	No effect
	Low level of senescence markers	Leiden 85-plus Study [52]	89.8 (85–101)	No dementia: 452 Dementia: 146 Incident dementia: unknown	Cognition Dementia Incident dementia	No effect
Markers of neurodegeneration	Normal levels of Aβ and tau in CSF	H85 Gothenburg study [56]	85 (85)	No incident dementia: 28 Incident dementia: 7	Incident dementia	Protective
	Negative amyloid PET-scan	The 90+ Study [57]	94.2 (90–99)^d^	No incident dementia: 10 Incident dementia: 3	Cognitive decline	Protective
	Less brain atrophy	H85 Gothenburg study [58]	85 (85)	No dementia: 30 Dementia: 23	Dementia	Equivocal
	Less WMH	H85 Gothenburg study [59]	85 (85)	No dementia: 133 Dementia: 103	Dementia	Protective
	High white matter integrity	The 90+ Study [60]	94.6 (90–103)	Normal: 64 CIND: 30	CIND	No effect

*Aβ* Amyloid β, *AD* Alzheimer’s disease, *APOE* Apolipoprotein E, *CIND* Cognitive Impairment, No Dementia, *CSF* cerebrospinal fluid, *DM* diabetes mellitus, *HDL* high-density lipoproteins, *LDL* low-density lipoproteins, *MCI* Mild Cognitive Impairment, *MMSE* Mini-Mental State Examination, *N* Number, *PET* positron emission tomography, *PLAD* Project of Longevity and Aging in Dujangyan, *WMH* white matter hyperintensities

^a^Mean age (range, if available, or ± if standard deviation) in years at baseline, unless stated otherwise; ^b^Median age (interquartile range, IQR) in years; ^c^Minimal and maximum mean age in years of the studies referred to; ^d^Median age (range) in years

Data about physical performance and activity have been collected in the Leiden 85-plus study and ***The 90+ Study***. Good physical performance, measured with handgrip strength, 4 m walk or standing balance tests, was associated with better cognitive functioning and lower dementia incidence in the oldest-old but high physical activity did not seem to influence dementia incidence [16, 40, 41].

With regard to genetics, the Apolipoprotein E (APOE) genotype, a major risk factor for AD in younger subjects, has been extensively studied in the oldest-old, with mixed results regarding the relation to cognition and dementia [42–46]. The Danish 1905 birth cohort, PLAD and Vantaa 85+ Study also studied a number of other genotypes in the oldest-old and found some additional protective and risk genotypes which are described in Table 2.

#### Hallmarks of aging and cognition in the oldest-old

Hallmarks of aging [47], such as inflammation and cellular senescence [48], have been scarcely studied in relation to cognition in the oldest-old. The Leiden 85-plus Study and ***The 90+ Study*** related inflammation markers to cognition and dementia but showed mixed results [49–51]. In addition, telomere length measured in white blood cells were not associated with cognition, dementia prevalence or incident dementia [52].

#### Markers of neurodegeneration and cognition in the oldest-old

Limited information is available about the relation of markers of neurodegeneration, such as amyloid β and tau measured in cerebrospinal fluid (CSF) and/or with a positron emission tomography (PET) scan with cognitive impairment in the oldest-old. Postmortem studies have shown that the prevalence of amyloid aggregation increases with age in cognitively healthy subjects but decreases in the oldest-old subjects with dementia [1]. A similar trend can be seen with regard to amyloid β measured in CSF or on an amyloid PET scan [53, 54]. In subjects without dementia, greater amyloid load has been associated with poorer cognitive functioning and a higher rate of incident dementia, although the number of oldest-old subjects in these studies was limited [55–57]. There are a few studies that have related brain MRI measurements in the oldest-old to cognitive functioning. Less atrophy and fewer white matter hyperintensities were seen in subjects without dementia compared to subjects with dementia [58, 59] but white matter integrity was not related to cognition [60]. In younger subjects, neurophysiological measures on magnetoencephalography (MEG) have been related to dementia [61] but it is unknown whether this relationship persists in the oldest-old.

### Aims and objectives of the EMIF-AD 90+ study

The EMIF-AD 90+ Study was set-up to investigate the protective factors for cognitive impairment in the oldest-old. The study was part of the Innovative Medicine Initiative (IMI) European Medical Information Framework for AD (EMIF-AD) project (http://www.emif.eu/about/emif-ad) on diagnostic markers, prognostic markers, and protective factors for AD. The EMIF-AD 90+ study focuses on the extreme phenotype of the cognitively normal oldest-old. The primary objectives of the EMIF-AD 90+ study are:

i) To identify factors associated with resilience to cognitive impairment in the oldest-old.
ii) To test the relationship between hallmarks of aging and cognitive impairment in the oldest-old.
iii) To test the relationship between markers of neurodegeneration and cognitive impairment in the oldest-old.

This paper describes the design and protocol of the study.

## Methods

### Study subjects

We aimed to include 80 cognitively normal subjects and 40 subjects with cognitive impairment, both aged 90 years and older. Inclusion criteria for cognitively normal subjects were a global Clinical Dementia Rating (CDR) score of 0 [62] and a score ≥ 26 points on the Mini-Mental State Examination (MMSE) [63]. Inclusion criteria for subjects with cognitive impairment were a diagnosis of amnestic MCI (aMCI) [64] or a diagnosis of probable or possible AD [65] by a neurologist, geriatrician, or general practitioner, a global CDR score ≥ 0.5 point (s) and a MMSE score of 20–28 points (inclusive). Exclusion criteria were the physical inability to undergo the procedures, visual or hearing impairment which made neuropsychological testing impossible, severe depression (Geriatric Depression Scale (GDS) score ≥ 11 points [66]) and other comorbidities or medication that could impair cognition at the discretion of the investigator (e.g. stroke, epilepsy or use of lithium carbonate). During the inclusion period it turned out to be difficult to identify subjects of 90 years and older with aMCI or probable or possible AD; we therefore broadened the inclusion criteria in this group to subjects older than 85 years.

Subjects were recruited at two sites: the Amsterdam UMC, The Netherlands and The University of Manchester, United Kingdom. Cognitively normal subjects were recruited from general practitioners or via advertisements (Amsterdam) or from the Manchester and Newcastle Ageing Study (MNAS, Manchester). Subjects with cognitive impairment were only recruited in the Netherlands. They were recruited from the Alzheimer Center Amsterdam and the Center Of Geriatric medicine Amsterdam (COGA) at the Amsterdam UMC, geriatric departments of other hospitals in the surroundings of Amsterdam, other healthcare facilities (such as a care home), general practitioners or via advertisement. The sample collection started on the 1st of June 2016 and ended on the 30th of June 2018. Currently we are working on the first data analyses.

The Medical Ethics Review Committee of the Amsterdam UMC approved the study in Amsterdam and the National Research Ethics Service Committee North West - Greater Manchester South performed approval of the study in Manchester. The study was carried out in accordance with the ethical conduct and juridical laws of the Declaration of Helsinki 64th WMA General Assembly, Fortaleza, Brazil, October 2013, (www.wma.net), and in accordance with the Medical Research Involving Human Subjects Act (WMO). All subjects gave written informed consent.

### Study design

The EMIF-AD 90+ Study is a case-control study in which we search for protective factors for cognitive impairment. Therefore, the cognitively normal subjects are described as cases and the subjects with cognitive impairment as controls.

### Study procedures

**Fig. 1:**
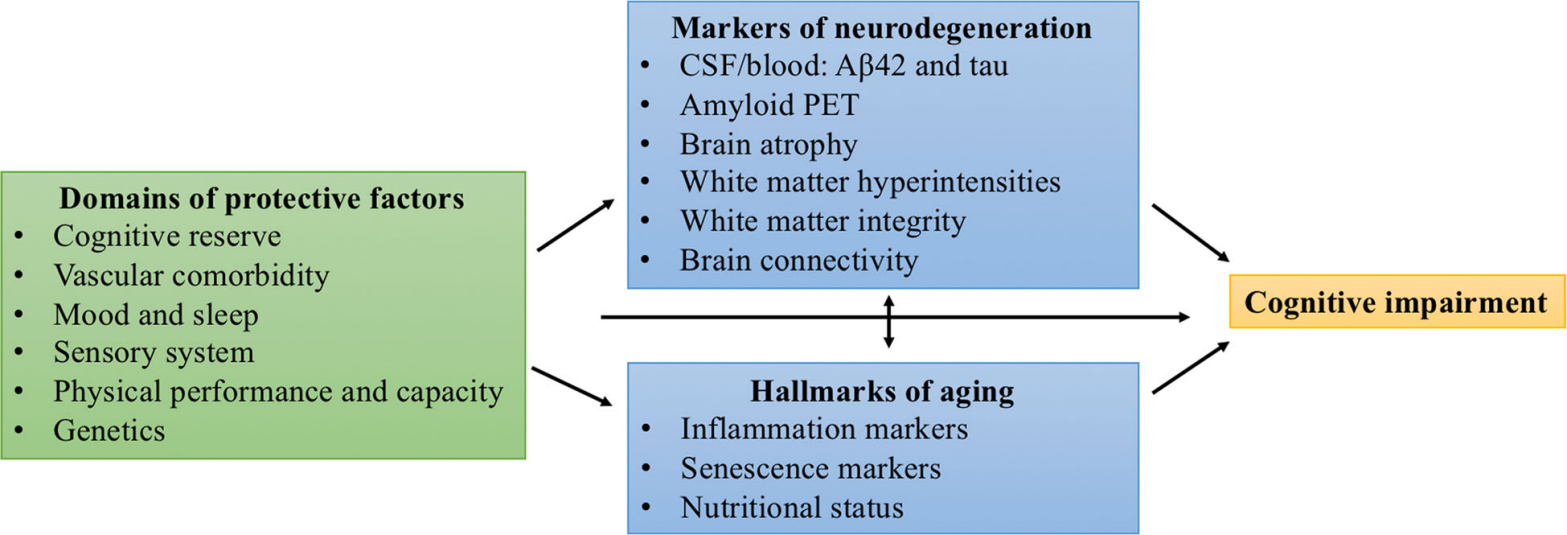
Overview of the domains of interest in the EMIF-AD 90+ Study. *Aβ* Amyloid β*, CSF* cerebrospinal fluid, *PET* positron emission tomography

The study consisted of two home visits and one or two visits at the hospital/clinical research facility (CRF). During the first home visit, in- and exclusion criteria (MMSE, CDR, impression of physical ability to undergo the procedures, hearing and visual abilities) were verified, in addition to collection of first study data (Table 3, paragraphs 2.3.1, 2.3.2 and 2.3.4). The MMSE is a short cognitive screening test with a maximum score of 30 points [63]. The CDR is a scale for the severity of symptoms of dementia, which was assessed by interviews with the subject and, if available, study partner (somebody that is in regular contact with the subject) in combination with judgement by the researchers [62]. The second home visit consisted of a neuropsychological assessment performed by a neuropsychologist (paragraph 2.3.3). During the hospital/CRF visits several procedures were performed, which are listed in Table 3 and described in paragraphs 2.3.4. – 2.3.10. These procedures provided information on i) potential protective factors (classified in six different domains), ii) hallmarks of aging, and iii) markers of neurodegeneration (Fig. 1 and Table 3). For each domain, hallmark of aging or markers of neurodegeneration, we will test one or more parameters (Table 3). In most cases, all procedures were performed within three months from start of the inclusion. Any differences in study procedures between Amsterdam and Manchester are explicitly stated in this paper.

**Table 3:** The domains of interest in the EMIF-AD 90+ Study

Domain	Parameter	Procedure (measurement)	Schedule Amsterdam	Schedule Manchester
Cognitive reserve	Level of education	Interview	Home	Home
	Cognitive activity	Cognitive abilities questionnaire	Home	Home
Vascular comorbidity	Cholesterol level, hypertension, DM, stroke, AF	Blood collection	Hospital	WMIC
		Medical history and medication use	Home	Home
		Blood pressure	Hospital	CRF
		Diagnostick/heart rate	Home	CRF
		Ultrasound carotid artery	Hospital	CRF
Mood and sleep	Depressive symptoms	Geriatric Depression Scale	Home	Home
	Sleep disorder	Berlin Questionnaire and MSQ	Home	Home
		Accelerometer (sleep quality)	Home	N/A
Sensory system	Visual acuity	ETDRS chart	Hospital	N/A
	Retinal thickness	OCT	Hospital	N/A
	Auditory function	Digits-in-noise test	Home	N/A
	Olfactory function	Sniffin sticks	Hospital	N/A
Physical performance and capacity	Physical performance	Grip strength	Home	CRF
		Short Physical Performance Battery or 4-min walking test	Hospital	CRF
		BIA (muscle mass)	Hospital	N/A
	Physical activity	Accelerometer	Home	N/A
Genetics	APOEε4 and APOEε2	Blood collection	Hospital	WMIC
Hallmarks of ageing	Level of inflammation markers	Blood collection (i.a. PBMCs)	Hospital	WMIC
	Level of senescence markers	Skin biopsy (senescence markers p16, p53 and telomere associated foci)	Hospital	N/A
	Nutritional status	BIA	Hospital	N/A
		Blood collection	Hospital	CRF
		BMI	Hospital	CRF
		MNA	Home	N/A
Markers of neurodegeneration	Aβ1–42 and tau in CSF and blood	CSF collection Blood collection	Hospital	N/A
	Amyloid PET scan	Amyloid PET scan	Hospital	WMIC
	Brain atrophy	Brain MRI scan or brain CT scan	Hospital	CRF
	WMH	Brain MRI scan or brain CT scan	Hospital	CRF
	White matter integrity	Brain MRI scan	Hospital	N/A
	Brain connectivity	Brain MRI scan	Hospital	CRF
		MEG	Hospital	N/A

*Aβ* Amyloid β*, AD* Alzheimer’s disease, *AF* atrial fibrillation, *APOE* Apolipoprotein E, *BIA* Bioelectrical impedance analysis, *BMI* Body Mass Index, *CRF* Clinical Research Facility, *CT* Computerized Tomography, *CSF* cerebrospinal fluid, *DM* diabetes mellitus, *ETDRS* Early Treatment Diabetic Retinopathy Study, *MEG* magnetoencephalography, *MNA* Mini Nutritional Assessment, *MRI* Magnetic Resonance Imaging, *MSQ* Mayo Sleep Questionnaire, *N/A* not applicable, *OCT* Optical Coherence Tomography, *PBMCs* Peripheral Blood Mononuclear Cells, *PET* positron emission tomography, *PLAD* Project of Longevity and Aging in Dujangyan, *WMH* white matter hyperintensities, *WMIC* Wolfson Molecular Imaging Centre

#### Interview

Data about the medical and family history, medication use, education and intoxications (alcohol use and smoking) were collected through a structured interview, in combination with information provided by the study partner (if available), general practitioner and/or medical specialist.

#### Questionnaires

In Amsterdam, subjects were asked to complete six questionnaires. Activities of daily living (ADL) were evaluated by use of the Katz ADL [67]. Functional health and wellbeing were evaluated by the Short form-12 Health-related Quality of Life (SF-12 HRQoL) questionnaire [68] and by the Cognitive Complaints Index (CCI) [69]. Nutrition was evaluated by the Mini Nutritional Assessment (MNA-long version) [70]. Sleep disorders were evaluated by use of the Berlin Questionnaire which identifies the risk of sleep disordered breathing [71]. Cognitive activity during life, such as reading books and playing games, was assessed with the cognitive abilities questionnaire [72]. Subjects with cognitive impairment filled in the questionnaires together with a study partner. The GDS was filled in together with the researcher [66].

In Amsterdam, the study partner was asked to complete five questionnaires: the AD8 (an 8-question test for the study partner to assess mild dementia) [73], the Amsterdam instrumental Activities of Daily Living (iADL) scale (a study partner based tool aimed at detecting iADL problems in early dementia) [74, 75], the Neuropsychiatric Inventory Questionnaire (NPI-Q, to assess the severity of behavioral symptoms in the subject and the distress these symptoms cause in the study partner) [76], the Mayo Sleep Questionnaire (MSQ, to screen for the presence of Rapid Eye Movement (REM) sleep disorders) [77], and finally the CCI [69].

In Manchester, subjects were asked to complete the SF-12 HRQoL questionnaire [68], the Physical Activity Scale for the Elderly (PASE) [78], the CCI [69] and the cognitive abilities questionnaire [72]. The study partner was asked to complete the AD8 [73], the Functional Activities Questionnaire (FAQ) [79] and the CCI [69].

#### Neuropsychological assessment

The neuropsychological assessment took approximately one and a half hours during which several cognitive domains were tested. Table 4 gives an overview of the different cognitive tests that were administered, which domain they examine and at which site they were performed.

**Table 4:** Cognitive tests in the EMIF-AD 90+ Study

Cognitive test	Cognitive domain	Site
CERAD 10 words test [115] Immediate recall Delayed recall after 10 min	Memory	B^a^
Logical Memory test [116] Immediate recall Delayed recall after 20–30 min	Memory	A
Rey Auditory Verbal Learning Test [117] Immediate recall Delayed recall after 20 min	Memory	M
Rey Complex Figure Test [118] Copy Delayed copy after 3 min	Memory Visuoconstructive skills	B
WAIS-III Digit span forward and backward [119, 120]	Executive functioning	B
Animal (2 min) and Letter fluency (1 min per letter^b^) [121]	Executive functioning	B
Clock Drawing Test^c^ [122]	Executive functioning Visuospatial functioning	A
Graded Naming Test [123]	Object-naming ability	B
Trail Making Test A and B [124]	Information processing speed Visual attention Task switching	B
WAIS-R Digit Symbol Substitution Test [125]	Perceptual-motor speed Incidental learning	B
Computerised Cambridge Neuropsychological Test Automated battery [126]	Paired associate learning Spatial-working memory Reaction time	B
National Adult Reading Test [127]	Pre-morbid IQ	B
Visual Association Test [128]	Visuospatial association learning	A
Addenbrooke’s Cognitive Examination Revised battery [129]	Attention/orientation Memory Verbal fluency Language Visuospatial abilities	M

*A* administered only in Amsterdam, *B* administered in Amsterdam and Manchester, *CERAD* Consortium to Establish a Registry for Alzheimer’s Disease, *M* administered only in Manchester, *min* minute (s), *WAIS (−R)* Wechsler Adult Intelligence Scale (-Revised)

^a^In Manchester only in the cognitively normal subjects. ^b^In Amsterdam using the letters D, A and T and in Manchester the letters F, A, and S. ^c^The subject will be asked to draw a clock showing the time “ten after eleven”. In total 14 points can be scored based on the presence and sequencing of the numbers and the positioning of the two hands

#### Physical examination

In Amsterdam, data on waist and hip circumference (cm), and hand grip strength (kg), as well as a standard neurologic screening examination were recorded during the first home visit. Hand grip strength was measured to estimate muscle strength and was performed with a hand dynamometer (Jamar hand dynamometer; Sammons Preston, Inc., Bolingbrook, IL., USA) [80]. In addition, a ‘Diagnostick’ was used to determine whether the subject had atrial fibrillation by measuring one derivative of an electrocardiogram [81]. At the end of the first home visit, the subject was asked to wear an accelerometer (DynaPort MoveMonitor, McRoberts B.V., The Hague, The Netherlands) for seven days to measure physical activity and sleep quality.

During the hospital visit in Amsterdam, continuous blood pressure measurements were performed non-invasively using a digital photoplethysmogram on the right middle finger (Nexfin®, BMEYE, Amsterdam, The Netherlands), resulting in beat-to-beat BP data. The Short Physical Performance Battery (SPPB) included balance tests, a 4 m walk to measure walking speed and the chair stand test [82]. Body composition, including the Body Mass Index (BMI), was measured using a Bioelectrical Impedance Analysis (BIA; InBody 770; Biospace Co., Ltd., Seoul, Korea).

In Manchester, waist and hip circumference (cm), hand grip strength (kg), BMI, resting blood pressure, heart rate, ankle/brachial pressure index [83] and a 4 min walking test were recorded at the clinical research facility.

#### Sensory system

Measurements of the sensory system were only performed in Amsterdam. With regard to visual functioning, best corrected visual acuity was tested with an Early Treatment Diabetic Retinopathy Study (ETDRS) chart. Intra-Ocular Pressure (IOP) and refraction data of all subjects were obtained, and all subjects underwent slit lamp examination and indirect fundoscopy. Pupils were dilated using tropicamide 0.5% and phenylephrine 5%. Peripapillary Retinal Nerve Fiber Layer (pRNFL) thickness and macular (layer) thickness were measured with Spectral Domain Optical Coherence Tomography (SD-OCT, Heidelberg Spectralis) using Heidelberg’s build-in software [84]. With enhanced depth imaging, the choroid was imaged and its thickness was (manually) measured. With fundus photography (Topcon TRC 50DX type IA), we acquired digital fundus images (50°). From these, seven Retinal Vascular Parameters (RVPs) were obtained using Singapore I Vessel Assessment (SIVA, version 3.0) [85].

For the auditory function, we used the digits-in-noise (DIN) test [86]. The DIN test is a speech-in-noise test using digit triplets as speech material. The digit triplets are presented against a constant level of stationary background noise. The test uses an adaptive procedure to determine the signal-to-noise ratio at which a listener understands 50% of the digit triplets correctly (i.e. the speech reception threshold (SRT) in noise). Olfactory function was measured using “Sniffin’ Sticks” (Burghart, Wedel, Germany). The test consists of pen-like odor dispensing devices with odors that are considered to be familiar. The smell test in the present study contained the odor identification part of the test [87].

#### Blood collection and skin biopsy

In both centers, blood samples were collected according to the biobanking pre-analytical Standard Operating Procedures (SOPs) of the Biomarkers for Alzheimer’s disease and Parkinson’s disease (BIOMARKAPD) project [88]. Blood samples were collected for DNA and RNA analysis, inflammation markers, proteomics, neurodegenerative markers (amyloid β, tau, neurofilament light), routine blood analysis (i.e. lipids and glucose), vitamin status (B12 and folic acid) and, in Amsterdam only, for Peripheral Blood Mononuclear Cells (PBMCs). Planned DNA analysis includes Single Nucleotide Polymorphisms (SNP) analysis of known genetic risk factors for AD or amyloid pathology [89–92]. DNA and RNA isolation will be performed by EMIF-AD partners. Remaining samples will be stored for future biomarker identification and validation studies.

In Amsterdam, four millimeter skin biopsies were taken from the inner upper medial arm and will be stained for senescence markers p16, p53 and telomere associated foci.

#### Cerebrospinal fluid collection

In Amsterdam, up to 20 mL CSF was obtained by lumbar puncture in Sarstedt polypropylene syringes using a Spinocan 25 Gauge needle in one of the intervertebral spaces between L3 and S1. A half mL CSF was immediately processed for leukocyte count, erythrocyte count, glucose, and total protein. The remaining CSF was mixed and centrifuged at 1300–2000 × *g* at 4 °C for ten minutes. Supernatants were stored in aliquots of 0.25–0.5 mL and frozen within two hours at − 80 °C and stored for future biomarker discovery studies. The processing and storing of CSF was performed according to the BIOMARKAPD SOP [88]. Amyloid β 1–42, total tau and phosphorylated tau 181 will be analyzed in a single batch. Remaining samples will be stored for future biomarker identification and validation studies.

#### Ultrasound carotid artery

At both sites, a duplex ultrasound scan of the carotid artery was performed. In Amsterdam, the right carotid artery was scanned to assess intima media thickness and distension using ArtLab software [93, 94]. In Manchester, left and right carotid arteries were scanned to determine velocity, vessel thickness, stenosis and plaques, rated according to the North American Symptomatic Carotid Endarterectomy Trial guidelines [95].

#### Brain MRI scan

Subjects underwent locally optimized brain MRI protocols including 3D-T1, fluid attenuated inversion recovery (FLAIR), susceptibility weighted imaging (SWI), diffusion tensor imaging (DTI) and resting state functional MRI (rs-fMRI). MRI scans were performed on Philips 3 T Achieva scanners. Additionally, in Manchester regional cerebral blood flow was measured by arterial spin labelling [96], but no DTI scan was acquired in Manchester. In Amsterdam, if a subject could not undergo the MRI scan, we considered a CT scan (Philips Ingenuity TF or Gemini TF camera). Scans will be analyzed locally and centrally by EMIF-AD partners using the Neugrid infrastructure if applicable (see Additional file 1).

#### Amyloid PET scan

[^18^F] Flutemetamol, a specific fibrillary amyloid β radiotracer, was used for the amyloid PET scans. In Amsterdam, [^18^F] flutemetamol was produced by General Electric (GE) Healthcare at the Cyclotron Research Center of the University of Liège (Liège, Belgium) and PET scans were performed using a Philips Ingenuity TF PET-MRI scanner (Philips Medical Systems, Cleveland, Ohio, USA) or, in case of a PET-CT scan, the Philips Ingenuity TF (Philips Medical Systems, Best, the Netherlands) or Gemini TF scanner (Philips Medical Systems, Best, the Netherlands). In Manchester, [^18^F] flutemetamol was produced at the Wolfson Molecular Imaging Centre (WMIC)‘s Good Manufacturing Practice radiochemistry facility using GE Healthcare’s FASTlab and cassettes and PET scans were performed using a High Resolution Research Tomograph (HRRT; Siemens/CTI, Knoxville, TN). In both centers, the emission scan was performed in two parts. First a 30 min dynamic emission scan was started simultaneously with a bolus intravenous injection of 185 MBq [^18^F] flutemetamol. The second part of the scan was performed from 90 to 110 min post injection. In Amsterdam, immediately before each part of the PET scan a T1-weighted gradient echo pulse MRI or low dose CT scan was obtained. This MRI or CT scan was used for attenuation correction of the PET scan. In Manchester, two seven minute transmission scans, one before the first emission scan and the other after the second emission scan, using a ^137^Cs point source were acquired for subsequent attenuation and scatter correction.

All [^18^F] flutemetamol scans were read visually as positive or negative. Additionally, we determined time activity curves for each region of interest with cerebellum grey matter as input function [97]. The dynamic data were analyzed on a voxel-by-voxel level using the Simplified Reference Tissue Model 2 (SRTM2) [98, 99]. Finally, we investigated tracer uptake by using a simplified method: the standardized uptake value ratio (SUVr, target to grey matter cerebellum SUV over 90–110 min pi) [100]. Variability in acquisition of amyloid PET scans were reduced by harmonizing acquisition protocols and will be reduced by adding it to the analyses as a covariate.

#### Neurophysiology

In Amsterdam, MEG was performed using a 306 channel whole-head system (Elekta Neuromag Oy, Helsinki, Finland). Eyes-closed and eyes-open resting-state MEG data were recorded with subjects in supine position inside a magnetically shielded room. We will use transformed time series [101] to extract spectral properties (relative band power and peak frequency) [102], and estimates of functional connectivity between brain regions, and metrics that characterize the topology of the functional brain networks [103, 104]. These analyses will be applied using Elekta’s beamformer software, and both in-house developed Matlab tools and BrainWave software (http://home.kpn.nl/stam7883/brainwave.html).

### Planned statistical analyses

For each parameter listed in Table 3, we will test with logistic regression models whether it is associated with resilience to cognitive impairment. In addition, linear regression models will be used to associate the same parameters with cognitive functioning in the total sample. Potential additional analyses include the identification of protective factors for abnormal AD biomarkers in the subsample of cognitively normal subjects and the identification of protective factors for cognitive impairment in subjects with a high risk, for example APOE ε4 carriers.

## Discussion

We described the design of the EMIF-AD 90+ Study that aims to unravel the factors associated with resilience to cognitive impairment in the oldest-old. An important additional value of the EMIF-AD 90+ Study compared to the previous studies is the extensive phenotyping of subjects, which includes data about cognitive reserve, vascular comorbidities, mood, sleep, sensory system capacity, physical performance and capacity and genetic risk factors. Furthermore, the EMIF-AD 90+ Study is one of the first studies that collects a broad range of markers of neurodegeneration in the oldest-old, including an amyloid PET scan, amyloid β and tau measured in CSF and blood and neurophysiological measures.

The EMIF-AD 90+ is the first study worldwide that combines data regarding the hallmarks of aging with markers of neurodegeneration. The process of aging and the incidence of aMCI and possible or probable AD are very much interrelated. Our study allows to test hypotheses such as that common risk factors and pathways drive both the aging process and development of cognitive impairment or AD. Another strength of the EMIF-AD 90+ study is that we use objective measures wherever possible, instead of using questionnaires. For example, physical activity and sleep quality were measured with an accelerometer in Amsterdam.

To conclude, the results of the EMIF-AD 90+ Study will provide an important contribution to the existing literature in many different ways. It will extend our knowledge on protective factors for cognitive impairment in the oldest-old and will determine how hallmarks of aging and markers of neurodegeneration relate to cognitive impairment in this specific age group.

## Additional file

Additional file 1: **Table S1.** Brain MRI scan analyses in the EMIF-AD 90+ Study. (DOCX 26 kb)

## Abbreviations

ACE-R: Addenbrooke’s Cognitive Examination Revised
AD: Alzheimer’s disease
ADL: Activities of daily living
Amsterdam UMC: Amsterdam University Medical Centers
APOE: Apolipoprotein E
Aβ: Amyloid β
BIA: Bioelectrical Impedance Analysis
BIOMARKAPD: Biomarkers for Alzheimer’s disease and Parkinson’s disease
BMI: Body Mass Index
CANTAB: Cambridge Neuropsychological Test Automated battery
CCI: Cognitive Complaints Index
CDR: Clinical Dementia Rating
CERAD: Consortium to Establish a Registry for Alzheimer’s Disease
COGA: Center Of Geriatric medicine Amsterdam
CSF: cerebrospinal fluid
DIN test: digits-in-noise test
DSST: Digit Symbol Substitution Test
DTI: diffusion tensor imaging
EMIF-AD: European Medical Information Framework for AD
ETDRS: Early Treatment Diabetic Retinopathy Study
FLAIR: fluid attenuated inversion recovery
GDS: Geriatric Depression Scale
GE: General Electric
GNT: Graded Naming Test
HRRT: High Resolution Research Tomograph
iADL: instrumental Activities of Daily Living
IMI: Innovative Medicine Initiative
IOP: Intra-Ocular Pressure
MEG: magnetoencephalography
MMSE: Mini-Mental State Examination
MNA: Mini Nutritional Assessment
MNAS: Manchester and Newcastle Aging Study
MSQ: Mayo Sleep Questionnaire
N/A: not applicable
NART: National Adult Reading Test
NPI-Q: Neuropsychiatric Inventory Questionnaire
OCT: Optical Coherence Tomography
PASE: Physical Activity Scale for the Elderly
PBMCs: Peripheral Blood Mononuclear Cells
PET: positron emission tomography
PLAD: Project of Longevity and Aging in Dujangyan
pRNFL: peripapillary Retinal Nerve Fiber Layer
RAVLT: Rey Auditory Verbal Learning Test
RCFT: Rey Complex Figure Test
REM: Rapid Eye Movement
rs-fMRI: resting state functional MRI
RVPs: Retinal Vascular Parameters
SD-OCT: Spectral Domain Optical Coherence Tomography
SF-12 HRQoL: Short form 12 Health-related Quality of Life
SIVA: Singapore I vessel Assessment
SNP: Single Nucleotide Polymorphisms
SOP: Standard Operating Procedure
SPPB: Short Physical Performance Battery
SRT: speech reception threshold
SRTM2: Simplified Reference Tissue Model 2
SUVr: standardized uptake value ratio
SWI: susceptibility weighted imaging
TMT: Trail Making Test
VAT: Visual Association Test
WAIS: Wechsler Adult Intelligence Scale
WMH: white matter hyperintensities
