# Supplementary material for: Resilience to cognitive impairment in the oldest-old: design of the EMIF-AD 90+ study
Source: BMC Geriatr. 2018 Nov 26;18:289. doi: 10.1186/s12877-018-0984-z (PMC6258163; doi:10.1186/s12877-018-0984-z)
Supplement: Supplementary file 1 — Table S1. Brain MRI scan analyses in the EMIF-AD 90+ Study. (DOCX 26 kb) [file 12877_2018_984_MOESM1_ESM.docx]

**Additional file 1**

**Table S1. Brain MRI scan analyses in the EMIF-AD 90+ Study**

| **Visual rating** | **Volumetric measurement**  (both whole brain and regional volumes) | **White matter Integrity** (both whole brain and regional measures)^a^ | **Functional connectivity** (both whole brain and regional patterns)^b^ |
| --- | --- | --- | --- |
| White matter hyperintensities  Microbleeds  Medial temporal lobe atrophy [1,2] | Hippocampal volume (LEAP) [3]  Cortical thickness (FreeSurfer)  Subcortical structures (FreeSurfer) | Fractional anisotropy  Mean diffusivity [4,5] | Synchronization likelihood  Modularity  Path length  Phase lag index [6, 7] |

*DTI* diffusion tensor imaging, *LEAP* Learning Embeddings for Atlas Propagation, *FSL* FMRIB Software Library, *rs-fMRI* resting state functional MRI, *TBSS* Tract-Based Spatial Statistics.

^a^Assessed using DTI scans, group differences will be quantified with TBSS as implemented in FSL; ^b^Assessed using rs-fMRI and ICA/C-paste by FSL.

**References**

[1] Fazekas F, Chawluk JB, Alavi A, Hurtig HI, Zimmerman RA. MR signal abnormalities at 1.5 T in Alzheimer’s dementia and normal aging. Am J Neuroradiol 1987;8:421–6.

[2] Scheltens P, Launer LJ, Barkhof F, Weinstein HC, van Gool WA. Visual assessment of medial temporal lobe atrophy on magnetic resonance imaging: interobserver reliability. J Neurol 1995;242:557–60.

[3] Wolz R, Aljabar P, Hajnal J V, Hammers A, Rueckert D, Initiative ADN. LEAP: learning embeddings for atlas propagation. Neuroimage 2010;49:1316–25.

[4] Clerx L, Visser PJ, Verhey F, Aalten P. New MRI markers for Alzheimer’s disease: a meta-analysis of diffusion tensor imaging and a comparison with medial temporal lobe measurements. J Alzheimer’s Dis 2012;29:405–29.

[5] Cui Z, Zhong S, Xu P, He Y, Gong G. PANDA: a pipeline toolbox for analyzing brain diffusion images. Front Hum Neurosci 2013;7.

[6] Damoiseaux JS, Rombouts SA, Barkhof F, Scheltens P, Stam CJ, Smith SM, et al. Consistent resting-state networks across healthy subjects. Proc Natl Acad Sci U S A 2006;103:13848–53. doi:10.1073/pnas.0601417103.

[7] Sanz-Arigita EJ, Schoonheim MM, Damoiseaux JS, Rombouts SA, Maris E, Barkhof F, et al. Loss of “small-world” networks in Alzheimer’s disease: graph analysis of FMRI resting-state functional connectivity. PLoS One 2010;5:e13788. doi:10.1371/journal.pone.0013788.
